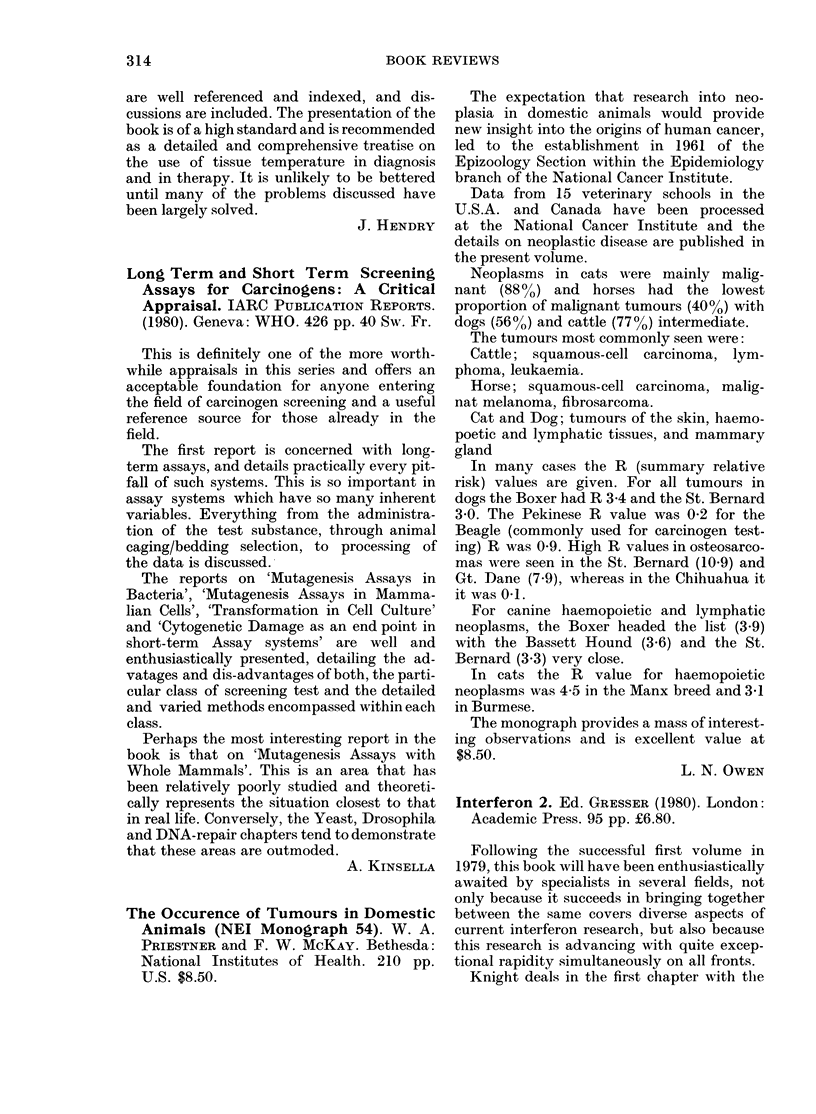# Long Term and Short Term Screening Assays for Carcinogens: A Critical Appraisal

**Published:** 1981-08

**Authors:** A. Kinsella


**Long Term and Short Term Screening Assays for Carcinogens: A Critical Appraisal.** IARC PUBLICATION REPORTS. (1980). Geneva: WHO. 426 pp. 40 Sw. Fr.

This is definitely one of the more worthwhile appraisals in this series and offers an acceptable foundation for anyone entering the field of carcinogen screening and a useful reference source for those already in the field.

The first report is concerned with long-term assays, and details practically every pitfall of such systems. This is so important in assay systems which have so many inherent variables. Everything from the administration of the test substance, through animal caging/bedding selection, to processing of the data is discussed.

The reports on 'Mutagenesis Assays in Bacteria', 'Mutagenesis Assays in Mammalian Cells', 'Transformation in Cell Culture' and 'Cytogenetic Damage as an end point in short-term Assay systems' are well and enthusiastically presented, detailing the advatages and dis-advantages of both, the particular class of screening test and the detailed and varied methods encompassed within each class.

Perhaps the most interesting report in the book is that on 'Mutagenesis Assays with Whole Mammals'. This is an area that has been relatively poorly studied and theoretically represents the situation closest to that in real life. Conversely, the Yeast, Drosophila and DNA-repair chapters tend to demonstrate that these areas are outmoded.

A. KINSELLA